# Stroke care in Germany: evidence gaps and priorities for health services research and quality assurance

**DOI:** 10.1186/s42466-026-00541-0

**Published:** 2026-10-30

**Authors:** Flora Noelle Wiegand, Dirk Bartig, Jürgen Faiss, Christoph Gumbinger, Stephen Kaendler, Tobias Knieß, Peter Kolominsky-Rabas, Felipe A. Montellano, Anne Lieb, Tobias Neumann-Haefelin, Christopher Jan Schwarzbach, Hassan Soda, Marco Stein, Inga Sünkeler, Götz Thomalla, Markus Wagner, Björn Misselwitz, Peter Heuschmann

**Affiliations:** 1 Institute for Clinical Epidemiology and Biometry, Julius-Maximilians-Universität Würzburg, Würzburg, Germany; 2 Institute for medical Data Science, University Hospital Würzburg, Würzburg, Germany; 3 DRG Market, Osnabrück, Germany; 4 German Stroke Society, Berlin, Germany; 5 Department of Neurology, University Hospital Heidelberg, Heidelberg, Germany; 6 Praxis für Nervenheilkunde, Offenbach, Germany; 7 Rhön Klinikum, Campus Bad Neustadt, Bad Neustadt an der Saale, Germany; 8 Interdisciplinary Center for Health Technology Assessment (HTA) and Public Health (IZPH), Friedrich-Alexander-University Erlangen-Nürnberg, Erlangen, Germany; 9 Department of Neurology, University Hospital Erlangen, Erlangen, Germany; 10 Department of Neurology, University Hospital Würzburg, Würzburg, Germany; 11 University Hospital Tübingen, Tübingen, Germany; 12 Klinikum Fulda, University Medicine Marburg – Campus Fulda, Fulda, Germany; 13 Neurologische Klinik, SRH Gesundheitszentrum, Bad Wimpfen, Germany; 14 Neurologische Klinik, Klinikum der Stadt Ludwigshafen, Ludwigshafen am Rhein, Germany; 15 Department of Neurosurgery, University Hospital Gießen, Gießen, Germany; 16 BDH-Klinik Braunfels, Braunfels, Germany; 17 Department of Neurology, University Hospital Hamburg-Eppendorf, Hamburg, Germany; 18 Stiftung Deutsche Schlaganfall-Hilfe, Eschborn, Germany; 19 Landesarbeitsgemeinschaft Qualitätssicherung Hessen, Eschborn, Germany

**Keywords:** Stroke, Health service research, Quality of care, Burden of disease

## Abstract

Stroke remains one of the leading causes of morbidity and mortality in Germany and worldwide. Within this position paper by the Commission Health Services Research and Quality Assurance of the German Stroke Society, we summarize current data on stroke epidemiology, healthcare delivery, and quality assurance of stroke in Germany, identify evidence and knowledge gaps across the stroke care pathway, and derive priorities for future health services research and quality assurance. Based on registry and routine administrative data, approximately 253,000 incident and recurrent strokes were estimated to occur in Germany in 2023. Although age-standardized stroke mortality rates had declined substantially since the 1980s, this trend has reached a plateau since 2018. Acute care is predominantly administered in certified stroke units, characterized by high process and outcome quality. However, areas for improvement remain in post-acute care and in structured outpatient aftercare, particularly regarding appropriate care for patients with persistent functional impairments and unmet needs. Future research priorities include for example the utilization of real-world data to document long-term outcomes, the expansion of quality assurance to specific entities such as subarachnoid hemorrhage, and the evaluation of telemedical frameworks in pre-hospital settings. Systematic implementation of Patient-Reported Outcome Measures (PROMs) in research and clinical practice could facilitate the identification of unmet needs and the development of patient-centered care models. In light of recent legislative advancements (e.g., the Health Data Use Act, European Health Data Space) and technological innovations (e.g., AI-driven applications), new opportunities are emerging to bolster health services research and further optimize the management of stroke patients.

## Introduction

Globally, stroke ranks as one of the leading causes of mortality and morbidity [1]. According to estimates from the Global Burden of Disease study, 11.9 million individuals experienced a stroke in 2021, accounting for 7.3 million deaths [1]. The following overview consolidates current data on the frequency and health care delivery of the disease condition stroke and identifies evidence gaps across the stroke care pathway in order to derive priorities for future health service s research and quality assurance in Germany.

## Epidemiology of stroke in Germany

The frequency of stroke is often estimated utilizing data from population-based registries. Since 1994, the Erlangen Stroke Registry has investigated stroke incidence across all age cohorts as part of the Federal Health Reporting program [2]. The mean age- and sex-specific incidence rates from 2016 to 2020 [3] derived from the Erlangen Stroke Registry were employed to estimate stroke frequency in Germany for the year 2023 (Table 1). In 2023, the mean annual age-standardized incidence rate for total stroke was 167 per 100,000 for women and 236 per 100,000 for men; for cerebral infarctions, the rates were 136 per 100,000 and 196 per 100,000, respectively. To estimate the absolute number of stroke patients in Germany in 2023, age- and sex-specific incidence rates were applied to the age and sex structure of the German population in 2023. The proportion of recurrent strokes was estimated based on data from the German Stroke Registers Study Group (Arbeitsgemeinschaft Deutschsprachiger Schlaganfall Register, ADSR) (Table 1). To estimate validity of this approach, the number of documented hospitalizations attributed to stroke (ICD-10: I60-I69) as well as to cerebral infarction (ICD-10: I63) as main diagnoses was reported in a standardized manner using nationwide routine data on hospital discharge based on § 21 claims data from the Institute for the Hospital Remuneration System (Institut für das Entgeltsystem im Krankenhaus, InEK) for 2023 [4] (Table 1).

**Table 1:** Estimated Stroke Cases in Germany in 2023

	Men	Women	Total
ESPro: Stroke			
First-time Stroke	98,869	91,797	190,314
Recurrent Strokes	31,393	29,148	60,429
Total Strokes	130,263	120,944	250,744
ESPro: Acute ischemic stroke			
First-time Cerebral Infarction	81,504	75,753	156,976
Recurrent Cerebral Infarction	25,879	24,053	49,843
Total Cerebral Infarction	107,383	99,806	207,189
G-DRG Data 2023: Cases coded as primary diagnosis (no duplicities)			
Total Strokes (ICD-10: I60, I61, I63, I64)	134,066	119,700	253,766
Total Cerebral Infarction (ICD-10 I63)	117,352	102,688	220,040

ESPro = Erlangen Stroke Project (Erlanger Schlaganfallregister). G-DRG = German Diagnosis-Related Groups; ICD= Intenational Classification of Disease; cases were identified based on ICD-coded primary diagnoses for 2023

**Fig. 1:**
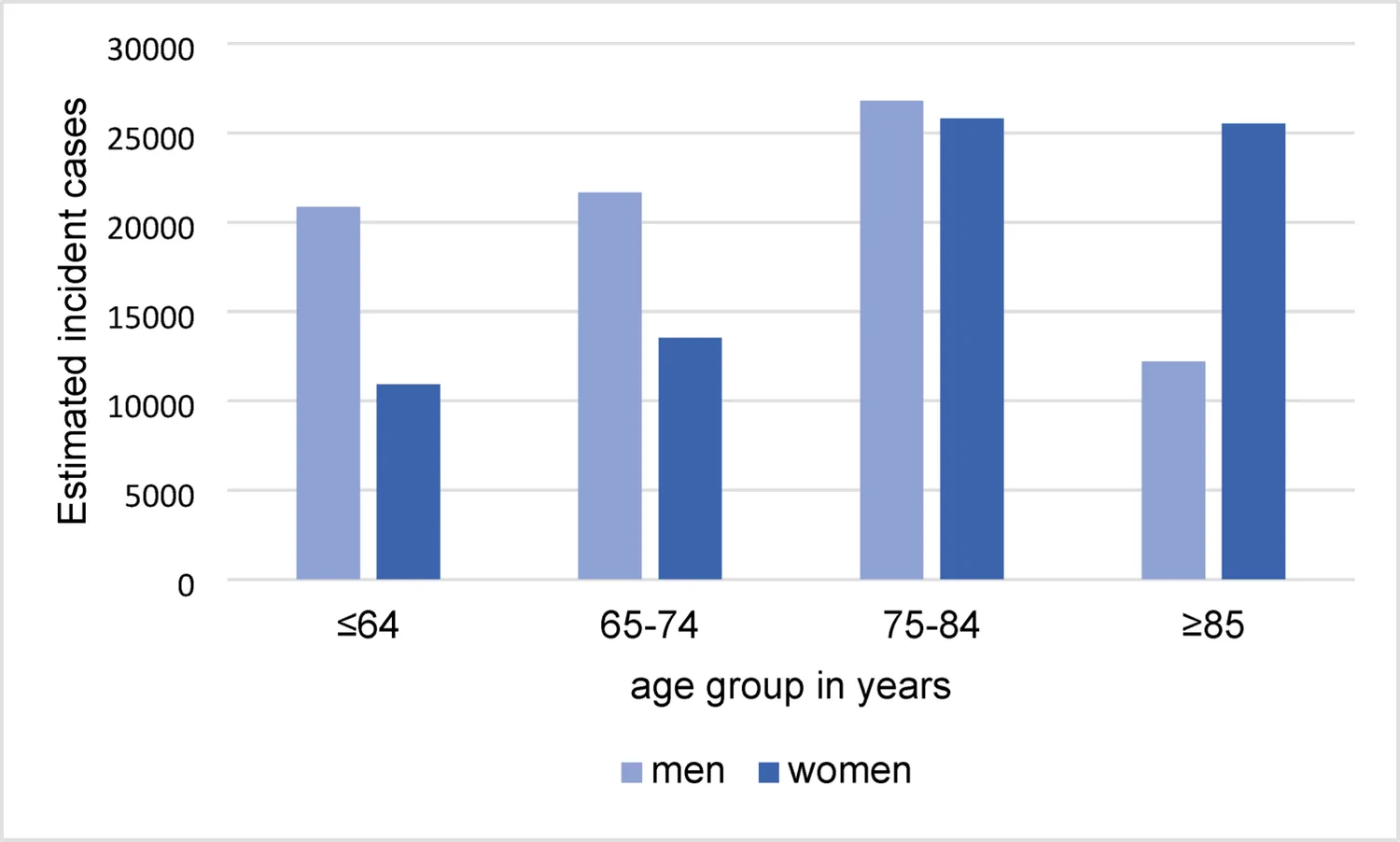
Estimated incident stroke cases by age and sex in 2023 based on data from the Erlangen Stroke Register (2016–2020)

Overall, the registry and the routine data approach estimated approximately 253,000 incident and recurrent strokes occurring in Germany in 2023 (Table 1). Notably, due to higher life expectancy, a greater absolute number of women than men experienced an initial stroke over age 85 (Fig. 1).

**Fig. 2:**
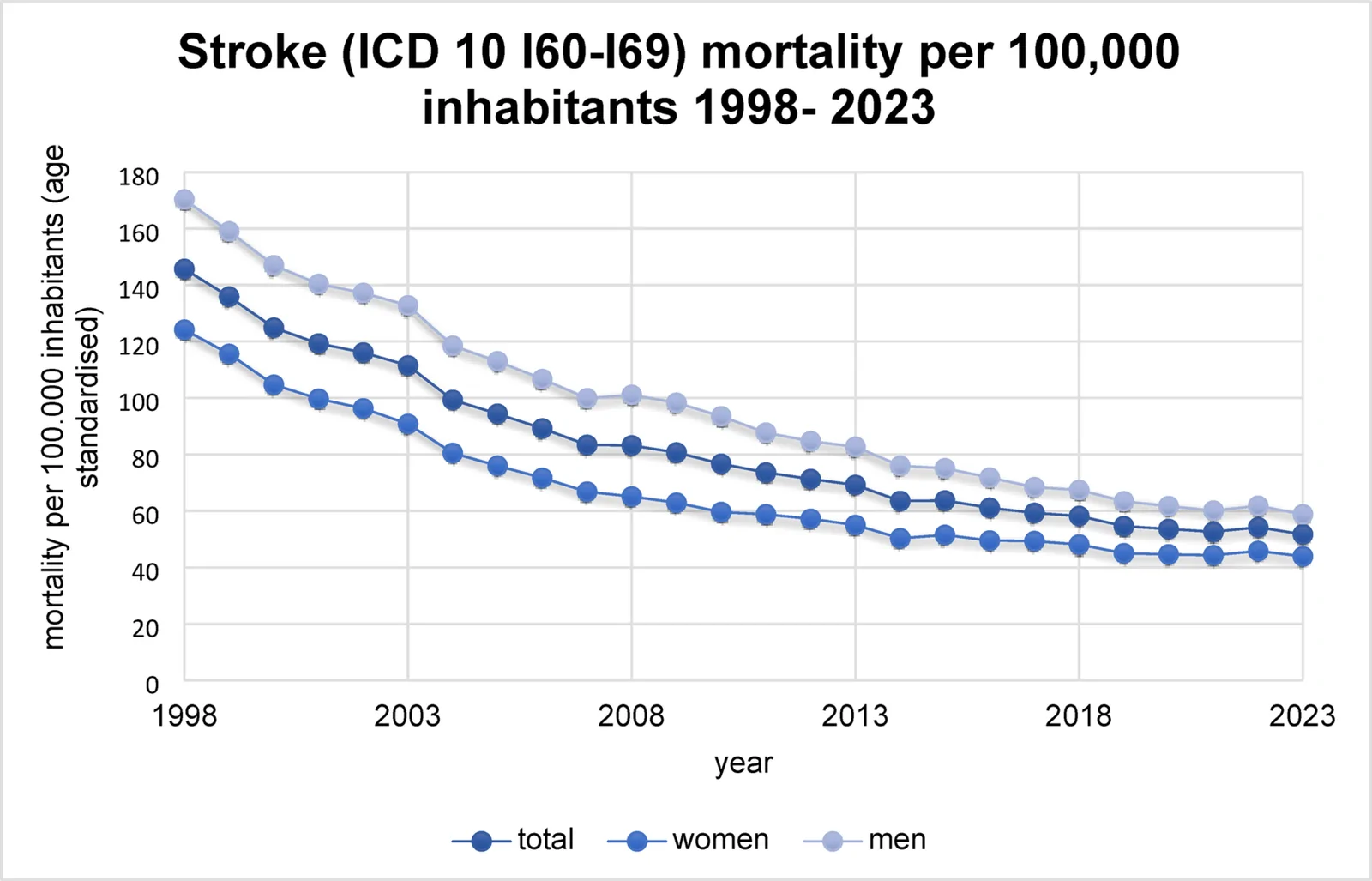
Development of stroke mortality (ICD-10: I60-I69) in Germany between 1998 and 2023 (Official cause of death statistics Germany 2023 (Adapted from Destatis [10]); age-standardized rates using the 2011 German standard population for men and women)

In 2023, stroke (ICD-10: I60-I69) accounted for approximately 5.2% of deaths in Germany [5]. Since the 1980s, age-standardized mortality rates have declined continuously [6]. From 1998 to 2015, age-standardized total mortality in both sexes decreased by more than 50%, particularly regarding cerebral infarctions [7]. However, a plateau can be observed since 2018 (Fig. 2). This trend is mirrored in other cardiovascular disease conditions with its causes remain unknown [8]. International projections anticipate a 30% increase in the absolute number of individuals living with long-term stroke-related disability by the late 2040s [9]. Currently, there is a lack of longitudinal projections regarding the future burden and epidemiological trajectory of stroke in Germany.

## Cost of stroke in Germany 2004–2025

Cost-of-Illness is strongly influenced by stroke subtype and stroke severity. Total costs are often reported from the perspectives of payers, healthcare providers, and society. The reported cost of stroke varies significantly due to divergent methods, different time periods, and data sources. Following international standards, costs are stratified by stroke subtypes. Reported costs represent the unified price level of 2018 [11]. However, available cost estimates are predominantly derived from studies based on data collected in the early 2000s, which may limit their generalizability to current stroke care.

- Cerebral Infarction (Ischemic Stroke): Total costs in the first year range between €15,137 and €22,666 [12–14]. Dodel and colleagues calculated the costs of cerebral infarctions in a Stroke Unit (SU) at €4,376 [15]. Lifetime direct costs after cerebral infarction, based on the Erlangen Stroke Project, amounted to €52,793 [13].
- Brain Hemorrhage (Intracerebral Hemorrhage (ICH)): Total costs for ICH in the first year are €23,886 [14]. Two studies examined acute costs in Germany, which ranged between €6,387 and €6,551 [15, 16].
- Subarachnoid Hemorrhage (SAH): Total costs for SAH in the first year of care range between €43,184 and €44,057 [15, 17].

## Quality of stroke care in Germany

### Pre-hospital and telemedical care

Tele-emergency physician systems are currently being implemented across several federal states to optimize pre-hospital emergency stroke management. Teleconsultations within emergency medical services have the potential to mitigate infrastructural challenges by facilitating the targeted deployment of emergency physicians. The availability of specialized expertise could enhance the management of specific emergency conditions, including suspected stroke. The forthcoming S2e guideline on telemedicine in pre-hospital emergency care advocates for the integration of specialized teleconsultation services [18], as basic neurological assessments for stroke can be performed with high diagnostic accuracy. Furthermore, large vessel occlusions (LVO) may be identified via telemedicine, facilitating expedited referral to endovascular-capable centers. Prospective evaluation of such approaches is warranted, as previous international randomized trials on scale-based triage have yet to demonstrate an advantage in functional outcomes at 90 days [19]. Nevertheless, such scales remain instrumental for direct referral protocols [20]. Moreover, the integration of new decision-making methods in the preclinical phase should be pursued and prospectively evaluated for example to distinguish patients with LVO from those with ICH who might particularly benefit from early interventions.

### Acute stroke management

In Germany, inpatient acute care for patients with stroke is predominantly delivered in acute care hospitals with a Stroke Unit. Routine data on the stroke complex treatment procedure allow a reliable classification of whether care was provided in a neurologist-led regional or supra-regional SU (German procedure classification (Operationen- und Prozedurenschlüssel (OPS) OPS 8-981.2 or 0.3) or in another type of SU with or without telemedical consultation (OPS 8-98b.2 or 0.3). Assessments from 2021 to 2022 estimate that approximately 90% of SU treatments are delivered in facilities with a neurology department, with slightly more than half taking place in supra-regional SUs. Telemedically linked SUs account for approximately 6% of complex stroke treatments and are required to be affiliated with a higher-level SU center [21].

**Table 2:** German stroke care structures based on certification levels by the German Stroke Society

Structure (DSG Certification)	Level of Specialization	Core Requirements and Treatment Capacities	Advanced Neurovascular Services	Function in Referral Pathways
Regional Stroke Unit (Regional SU)	Certified primary stroke center	4–10 monitored stroke beds. Dedicated stroke unit with continuous monitoring, stroke-trained multidisciplinary team, 24/7 CT imaging, intravenous thrombolysis, standardized diagnostics and secondary prevention.	Not required on-site. Access to neurosurgery, vascular surgery, and neuroradiology through formal cooperation agreements.	First-line specialized stroke care for a regional population. Transfers patients requiring mechanical thrombectomy, neurosurgical interventions, or highly specialized diagnostics to a supra-regional SU.
Supra-Regional Stroke Unit (Überregionale SU)	Highest level of DSG Stroke Unit certification	8–20 monitored stroke beds. All requirements of a Regional SU plus higher case volumes, broader neurological expertise, expanded intensive care capabilities, and 24/7 availability of advanced neurovascular diagnostics and treatment pathways.	Endovascular therapy (including mechanical thrombectomy), neuroradiology, neurosurgery, and other neurovascular specialties must be available on-site or with immediate availability.	Regional referral hub for complex cerebrovascular cases.
Comprehensive Stroke Unit	Additional certification for highly specialised centres		Full spectrum of neurovascular diagnostics and therapies, often including advanced neurocritical care and research infrastructure.	
Telemedicine-Linked Stroke Unit (Tele-SU)	DSG-certified Stroke Unit integrated into a telemedicine network	Local stroke care including emergency imaging, monitoring, and intravenous thrombolysis supported by 24/7 teleconsultation with stroke specialists at a network centre. Particularly designed for hospitals in rural or underserved regions.	Advanced interventions generally not available locally and require transfer to a supra-regional centre.	Extends access to guideline-concordant stroke care where on-site vascular neurologists are unavailable. Facilitates rapid triage and transfer of patients eligible for thrombectomy or other advanced treatments.
Neurovascular Network (NVN)	Separate DSG certification for network structures	Collaboration of multiple hospitals to link SUs, comprehensive SUs, neurosurgery, neuroradiology, vascular surgery, rehabilitation providers, and emergency medical services. Shared quality assurance and treatment standards.	Coordinated access to the full spectrum of neurovascular diagnostics and treatments across participating institutions.	Organizes transfers, expertise sharing, quality assurance, and regional access to highly specialized neurovascular care.

**Fig. 3:**
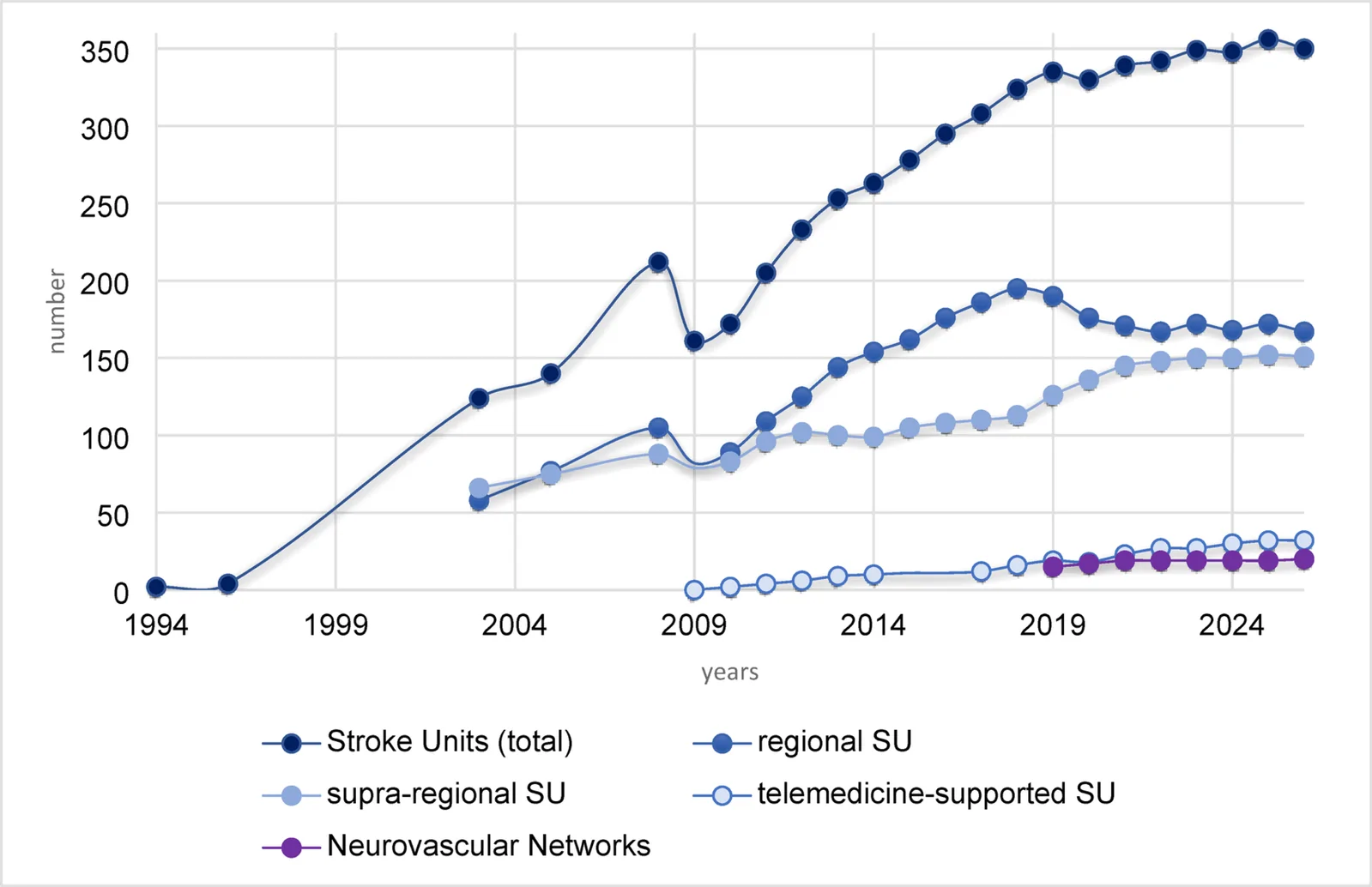
Certified Stroke Units and neurovascular networks in Germany since 1994

Although the reported figures on SU care refer to all patients with stroke, patients with ICH and subarachnoid hemorrhage (SAH) are only partially covered. SAH represents a distinct treatment scenario at the interface of care in neurological and neurosurgical departments, requiring timely diagnosis and intervention to optimize survival and functional outcomes.

Currently, SAH is not documented with a SAH specific dataset in stroke registries in Germany, with the exception of the federal states of Hesse and Bavaria [22]. Establishing a nationwide, SAH-specific quality assurance program would represent an important step towards systematically improving the quality of care for this patient population. 

At present, 352 SUs are certified in Germany (as of June 1st, 2026), including 152 supra-regional, 168 regional, and 32 telemedically linked certified SUs [23]. In addition, there are currently 19 certified neurovascular networks (as of June 1st, 2026). According to estimates by the German Stroke Society, the majority of facilities that meet the minimum case volume required for certification (approximately 85–90%) are currently being certified according to the criteria of the German Stroke Society’s stroke unit certification standards [24]. The established certification process evaluates quality structures, process, and outcome and requires participation in an external quality assurance program, thereby fulfilling the prerequisites for inclusion in the Hospital Atlas [25]. More than 20 years ago, an external nationwide quality assurance system was established in Germany by the German Stroke Registers Study Group (ADSR), which documents the quality of acute inpatient stroke care using evidence-based quality indicators developed in a standardized manner (Table 2) [26]. While the ADSR registries provide comprehensive and clinically detailed data on acute stroke care in Germany, the quality assurance data are primarily based on self-reported documentation from staff of the participating hospitals without a comprehensive source data monitoring. Consequently, the possibility of reporting bias, regional heterogeneity in documentation practices, and residual inaccuracies in selected variables cannot be completely excluded.

In 2023, the reference ranges for nearly all quality indicators were within the defined national target values and demonstrated a continuous improvement over time (Table 3). The external quality assurance program by the ADSR enables the identification of outlier values at individual hospitals, allowing targeted investigation of underlying causes and remediation of deficiencies, in some federal states also through a structured dialogue process [27]. A key research priority for quality assurance documentation is the increased use of external health care data and real-world data (RWD) (e.g., derived from electronic health records, claims data, or registries). This is of particular relevance in light of emerging legislative frameworks in Germany and the European Union, including the Health Data Use Act (Gesundheitsdatennutzungsgesetz, GDNG), the European Health Data Space (EHDS), and the planned registry law. Experiences from the Medical Informatics Initiative (MII) and the Network University Medicine (NUM) can be leveraged to assess the expanded use of routine care data, including for the evaluation of long-term outcomes [28]. Although the data infrastructure on stroke care in Germany is substantial, comprehensive analyses of the impact of regional care characteristics on processes and outcomes after stroke (e.g., the role of case volumes, tele-stroke services, and rural versus urban regions) are still lacking.

**Table 3:** Quality indicators for acute stroke treatment in 2023 within the ADSR registries*

	ID	Quality Indicator	Result %	Reference Range %**
Diagnostics	15	Treatment in a stroke unit	93.9	>=90
	13a	Initial brain imaging within 30 min after admission in patients with cerebral infarction and symptom onset ≤ 6 h	70.9	>=60
	13c	CT/MR/DS angiography following native imaging	94.4	>=70
	9	Vascular diagnostics within 24 h after admission in patients with TIA or cerebral infarction	93.5	>=80
	12	Screening for dysphagia	95.0	>=90
	22	Atrial fibrillation diagnostics	95.2	>=90
Acute Therapy	14d	Systemic thrombolysis (age ≤ 80, NIHSS 4–25, onset-to-admission time ≤ 4 h)	69.5	>=60
	16a	Door-to-needle time < = 60 min (Systemic thrombolysis, NIHSS 4–25, onset-to-admission time ≤ 4 h)	88.2	>=90
	23	Intra-arterial therapy (in case of vessel occlusion, (onset-to-admission time ≤ 6 h))	69.7	>=60
	25	Door-to-puncture time < = 90 min (Intra-arterial-therapy)	66.4	>=50
	27	Achievement of the recanalization target after intra-arterial therapy (TICI IIb–III)	87.7	>=80
Rehabilitation and Secondary Prevention	2	Physiotherapy/occupational therapy (modified Rankin Scale ≥ 3 or Barthel Index ≤ 70) no later than day 2 after admission	96.4	>=90
	3	Speech therapy (aphasia/dysarthria/dysphagia) no later than day 2 after admission	95.6	>=90
	17	Carotid revascularization in symptomatic carotid stenosis (Stenosis > = 70% and < 100%, Rankin Scale at Discharge < = 3 )	84.3	>=80
	6	Anticoagulation for atrial fibrillation at discharge	95.0	>=90
	21	Statin therapy at discharge	95.3	>=90
	19	Discharge to rehabilitation for patients with disabilities relevant to activities of daily living	77.9	>=70

*The results from the following registries were merged: Baden-Württemberg, Bayern, Berlin, Hamburg, Hessen, Nordwestdeutschland, Rheinland-Pfalz und Schleswig-Holstein. Together, they represent at least 80% of the strokes treated in Germany

**Established within the framework of a consensus reached by the DSG (German Stroke Society) and the ADSR in 2023

### Neurological post-acute rehabilitation

Despite the continuous improvements in pre-hospital and acute clinical care for stroke patients in Germany, an increasing structural deficit of rehabilitative follow-up treatment is emerging. In the field of neurology, the rehabilitation framework is categorized according to a ‘Phase Model’: Phase B is defined by a Barthel Index (BI) of < = 30, Phase C by a BI of > 30, and Phase D by a BI of ≥ 65 (under Statutory Health Insurance) or ≥ 75 (under German Pension Insurance).Currently, a significant discrepancy exists between the intensity required for rehabilitation treatment of severely impaired Phase C patients and the reimbursement provided through individually negotiated per-diem rates by the payers, making such care increasingly non-cost-effective. In contrast, the Diagnosis-Related Group (DRG) reimbursement for Phase B treatment is generally adequate with the actual resource expenditure, particularly in cases involving weaning. This economic imbalance has led to a strategic reduction in Phase C bed capacities in favor of expanding Phase B facilities in Germany. Consequently, there is a critical shortage of rehabilitation options for severely affected Phase C patients (BI < 50). At the same time, a substantial and potentially increasing proportion of stroke survivors are treated in geriatric hospitals providing geriatric early rehabilitative complex treatment or in geriatric rehabilitation facilities. This trend may partly reflect limited capacities and restrictive access to neurological Phase C rehabilitation facilities and highlights an important structural issue regarding the allocation of patients between neurological and geriatric rehabilitation pathways. The impact of these different rehabilitation trajectories on functional outcomes, long-term participation, and healthcare utilization warrants further investigation in future health services research projects. To ensure comprehensive stroke management, it is important to implement a seamless, needs-oriented neurological follow-up rehabilitation trajectory after acute stroke care.

### After care

In contrast to other European countries, comprehensive, structured outpatient after care services are lacking in Germany [29–31]. While a Disease Management Program (DMP) for coronary heart disease was successfully implemented in 2003 [32], a comparable framework for stroke patients in Germany remains absent. Various healthcare projects, partially funded by the Innovation Fund of the Federal Joint Committee (Gemeinsamer Bundesausschuss, G-BA), focusing on cross-sectoral, evidence-based, and systematic aftercare have demonstrated improvements in cardiovascular risk profiles or functional status following an acute event. However, these initiatives failed to show a significant reduction in cardiovascular events or a substantial improvement in health-related quality of life [33, 34], leading to a negative appraisal of ambulatory stroke follow-up projects by the G-BA. Notably, in the StroCare project, patients in the intervention group exhibited superior functional outcomes as measured by the modified Rankin Scale (mRS) [34]. The subsequent evaluation of long-term effects, such as in the SANO-EXTEND project, may contribute to a reassessment of these services in the future [35]. Furthermore, since 2024, the LEX LOTSEN OWL project has been supported to facilitate the integration of patient navigators into standard care [36]. Regardless of the lack of a legal frameworks, numerous regional care initiatives and also digitally-supported stroke follow-up concepts exist in Germany [37, 38]. Studies on the case-management-based SOS-Care project and the Austrian StroCard project indicate a reduction in the combined endpoint of vascular death and stroke compared to routine care [31, 39]. These studies were conducted at few highly specialized centres, thus, their success may be attributed to the careful implementation and control of complex care interventions. Consequently, Austria legally established structured ambulatory after care in the form of a mandatory three-month follow-up visit in 2022. Comparable regional after care programs have also been implemented in other European countries: in the Netherlands, the cluster-randomized ECO-stroke trial evaluates a nurse-led, patient-tailored screening and referral intervention [29]; in Sweden, the nurse-led, telephone-based NAILED trial demonstrated sustained improvements in vascular risk factors, including blood pressure and LDL cholesterol, at 36-month follow-up [30]. Potential future health services research projects in the field of after care should align with actual care requirements to address the heterogeneous needs of the patient collective (e.g., patients with severe stroke, those with high cardiovascular risk profiles or with specific sequelae). Beyond recurrent vascular events, stroke causes a substantial long-term burden through different sequela such as cognitive impairment, depression, anxiety, fatigue, communication disorders, or restrictions in daily activities and social participation. Family caregivers are also frequently affected by considerable psychosocial burden. The heterogeneous care pathways after discharge, involving rehabilitation facilities, outpatient specialists, primary care physicians, therapists, and long-term care services, often result in unmet needs. The German Stroke Society (DSG) Aftercare Commission addresses the fragmentation of current aftercare structures in Germany and advocates for structured, multidisciplinary, and patient-centered long-term follow-up [40–42].Furthermore, using Patient-Reported Outcome Measures (PROMs) appears more appropriate as primary endpoint for evaluating the multifaceted impact of interventions, as exemplified in successful applications in heart failure projects [43].

### Nursing

**Fig. 4:**
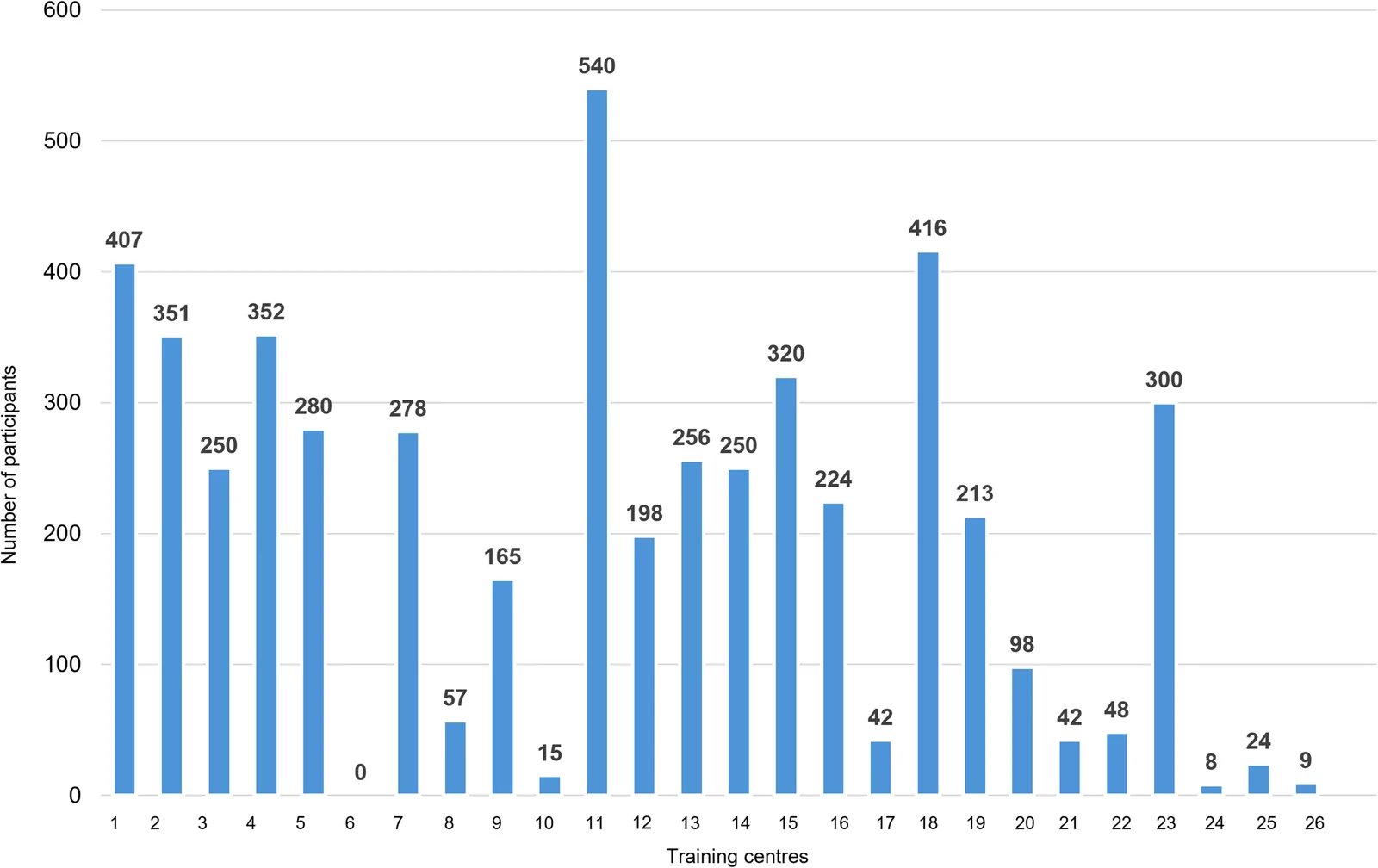
Total number of Stroke Nurses trained between 2006 and 2024 per training center (*n* = 5,150)

Successful management of stroke patients requires an interdisciplinary collaboration between physicians, nursing staff, and the therapeutic team across the entire continuum of care. Since 2006, the DSG has established a specialized training curriculum for nursing staff at SU, leading to the “Specialized Stroke Unit Nursing/Stroke Nurse” qualification. Obtaining SU certification is now contingent upon maintaining a mandatory minimum number of qualified Stroke Nurses on each ward. In recent years, a nationwide network of training centers has emerged in Germany, currently comprising 27 locations with two conducting a joint training centre. Approximately 480 specialists are qualified for SU service annually, with the number of centers expanding in response to the increasing clinical demand. The Stroke Nurse program has expanded substantially since its introduction in 2006, resulting in more than 5,000 certified Stroke Nurses by 2024, with substantial variation in training activity (see Fig. 4). However, the impact of specialised Stroke Nurse training on health care processes and patient outcomes has not yet been systematically evaluated.

Notably, the curriculum’s scope is currently limited to the acute phase on the SU rather than a distinct qualification comprising post-acute rehabilitation or long-term stroke after care. Given the growing recognition of the entire care continuum’s role in stroke outcomes, an explicit extension of training activities complementing the current focus on acute-care qualification might be a valuable area for future development. A nationwide scientific evaluation covering all SU would be highly desirable to analyze the efficacy of this advanced specialized training in stroke management compared to earlier standard care models.

### Needs and requirements from the patient perspective

Value-Based Health Care (VBHC) has gained increasing importance since the mid-1990s, integrating Patient-Reported Outcomes (PROs) as a fundamental metric for evaluating therapeutic interventions. The utilization of PROs is regarded as a highly promising endpoint to improve both the quality and the efficiency of healthcare delivery. In the context of stroke management, PROs are also increasingly used to assess outcomes within the clinical and the research context [44]. To facilitate this, the International Consortium for Health Outcomes Measurement (ICHOM) has developed a standardized outcome set that incorporates also specific Patient-Reported Outcome Measures (PROMs) [45]. However, despite their anticipated potential, PROMs are rarely utilized as primary endpoints in the clinical or the research context, and a systematic framework for capturing the perspectives of stroke survivors needs to be established [46]. The EPOS project (Outcome Quality through PROMs), funded by the G-BA Innovation Committee, has recently confirmed high levels of patient acceptance regarding the use of these instruments in Germany [47]. In this context, digital tools represent a promising implementation strategy for identifying individual patient needs and collecting PROMs efficiently [48]. Stroke survivors in the post-acute phase exhibit a wide spectrum of unmet needs, ranging from specific health-related requirements to challenges in social participation [49]. A meta-analysis examining the long-term sequelae of stroke revealed that neuropsychiatric symptoms including anxiety, depression, fatigue, and sleep disorders affected 26% to 60% of patients up to ten years post-event [50]. Integrating PROMs into existing quality assurance projects and routine clinical documentation in addition to supplementary surveys of patients would identify unmet needs across the entire care continuum and could provide critical insights for defining future research priorities.

## Future perspectives

A variety of emerging developments facilitate a more comprehensive description of health care for stroke patients and allow to implement measures for further optimizing stroke patient management in Germany. These developments include:

- Enhanced data utilization: Broader availability, simplified access, and improved usability of RWD facilitated by new legislative frameworks. This encompasses the linkage of different data sources and as well as the application of innovative methodological approaches, such as comparative effectiveness research for medical treatments, or the development, implementation, and evaluation of complex interventions.
- Integration of patient perspectives: An increasing recognition of the clinical and scientific value of incorporating patient perspectives into shared decision-making and in the evaluation of interventions within both, the therapeutic and study contexts.
- Integration of stroke care provider perspectives: Research addressing the perspectives, resources, and structural barriers of health care professionals delivering long-term post-stroke care, e.g., general practitioners and community- or office-based neurologists, is needed to identify obstacles to implementation and to inform sustainable, scalable models of aftercare.
- Technological innovation: The integration of digital health technologies, telemedicine, and AI-supported applications, e.g., for enabling the systematic documentation of PROs and the delivery of individualized follow-up care.
- Collaborative research infrastructures: The establishment of multicenter networks for collaborative clinical research, alongside the development of standardized tools and datasets for the secondary use of clinical data in Germany (e.g., the Study Network Stroke within the Network University Medicine (NUM), as well as core datasets and the ‘broad consent’ framework of the Medical Informatics Initiative (MII)).
- Funding opportunities: Expanded funding opportunities specifically targeted at performing, evaluating and implementing interventional health services research projects within the German healthcare system.

## Conclusion

Stroke management in Germany has a high standard especially during the acute phase, primarily attributable to the nationwide coverage of SU and the implementation of quality assurance measures. Conversely, significant deficits exist in rehabilitative after care, SAH specific management, and the utilization of RWD for assessing long-term outcomes. Going forward, individualized after care concepts, telemedical solutions, and patient-centered endpoints, such as PROMs, need to be more robustly integrated into routine care. Structured health services research projects can provide targeted support for these developments, ultimately enhancing the long-term quality of care. The following priority research themes have been identified for future projects aimed at optimizing stroke care in Germany:

- **Quantifying the long-term burden of stroke**: Generating projections regarding the long-term epidemiological trajectories and impact of stroke in Germany, as well as identifying drivers of temporal trends through the integration of different data sources and high-quality longitudinal real world and electronic health record data.
- **Evaluating and optimizing pre-hospital care pathways**: Evaluating novel concepts to improve pre-hospital management and the referral protocols for stroke patients.
- **Improving SAH-specific quality of care**: Enhancing quality assurance frameworks with SAH specific metrics to delineate potential improvements at the interface between neurology and neurosurgery.
- **Evaluating regional health care structures**: Investigating the impact of regional care infrastructures on clinical processes and patient outcomes.
- **Developing and evaluating individualized aftercare programs**: Evaluating the impact of individualized supportive measures in stroke aftercare, as well as developing specialized care concepts for specific stroke subgroups using routine and electronic health record data.
- **Defining and implementing patient-centered outcomes**: Defining alternative endpoints for patient-centered measurement and supplementing existing quality assurance and routine documentation with the longitudinal assessment of PROMs throughout the entire care pathway.

## Data Availability

The data supporting the findings of this study were provided in anonymized form by the German Stroke Registers Study Group (ADSR). Due to data protection regulations, the data are not publicly available but may be obtained from the respective ADSR registers upon reasonable request and subject to approval by the regulations of the respective data-holding institution. In addition, this study used aggregated data from the Diagnosis Related Groups (DRG) statistics (§21 Krankenhausentgeltgesetz (KHEntgG)) provided by Destatis (Federal Statistical Office of Germany). These data must be requested from Destatis.
